# Cultivating Patient Preferences in ALS Clinical Trials: Reliability and Prognostic Value of the Patient-Ranked Order of Function

**DOI:** 10.1212/WNL.0000000000209502

**Published:** 2024-06-14

**Authors:** Ruben P.A. van Eijk, Leonard H. van den Berg, Ying Lu

**Affiliations:** From the UMC Utrecht Brain Center (R.P.A.v.E., L.H.v.d.B.), University Medical Center Utrecht, the Netherlands; and Department of Biomedical Data Science (Y.L.), Stanford University, CA.

## Abstract

**Background and Objectives:**

The Patient-Ranked Order of Function (PROOF) is a novel approach to account for patient-reported preferences in the evaluation of treatments of amyotrophic lateral sclerosis (ALS). In this study, we assess the reliability and prognostic value of different sets of patient-reported preferences that can be used for the PROOF end point.

**Methods:**

Data were obtained through online surveys over the course of 12 months using the population-based registry of the Netherlands. Patients were asked to score functional domains of the ALS Functional Rating Scale (ALSFRS-R) and rank the order of importance of each domain. Two weeks after the initial invite, the questionnaire was repeated to evaluate test-retest reliability. Vital status was extracted from the municipal population register.

**Results:**

In total, 611 patients with ALS were followed up for survival and 382 patients were included in the test-retest reliability study. All versions of PROOF, using different sets of preferences, resulted in excellent reliability (intraclass correlation coefficients ranged from 0.89 [95% CI 0.87–0.91] to 0.97 [95% CI 0.97–0.98], all *p* < 0.001), without systematic differences between baseline and week 2 (mean rank difference range −1 to −3 [95% CI range −8 to 2], all *p* > 0.20). Preferences about future events were more variable than preferences about current symptoms. All versions of PROOF strongly predicted overall survival (hazard ratios per 10th rank percentile ranged from 0.80 to 0.83 [95% CI range 0.76–0.87], all *p* < 0.001) and had a more even separation of survival curves between rank-stratified subgroups compared with the ALSFRS-R total score.

**Discussion:**

In a large cohort of patients, we show how patient-reported preferences can be measured and integrated reliably with the ALSFRS-R without leading to systematic bias. Patient preferences may provide unique prognostic information in addition to what is already measured conventionally. This could provide a more comprehensive understanding of how medical interventions effectively address the patient's concerns and improve what matters most to them.

## Introduction

Growing patient advocacy has led to increasing interest in patient-focused drug development,^1,2^ and this is also gaining momentum among patients with amyotrophic lateral sclerosis (ALS).^3^ Recently, several guidance documents were released by major regulatory agencies to make patient experience of disease and treatment an integral part of drug development and regulatory decision making.^4,5^ This information is critical because statistical significance does not, in itself, indicate whether an observed effect is clinically meaningful. This becomes even more important for diseases such as ALS with variable clinical manifestations that affect patients differently^6-8^: The value of a treatment really depends on how the symptoms—regarded by patients as most important—are improved.^9,10^

Hence, it will be critical for patient-focused approaches to recognize and incorporate heterogeneity in patient preferences in outcome evaluations of new treatments. For this purpose, the Patient-Ranked Order of Function (PROOF) has been developed.^10,11^ PROOF weighs the treatment benefit according to the symptomatic domains that are most important to patients. The methodology underlying PROOF is based on prioritizing outcomes, sharing similarities with other composite end points, such as the desirability of outcome ranking or the win ratio.^12,13^ The main difference between these end points and PROOF is that outcomes are prioritized based on patient-reported preferences rather than clinical significance, with prioritization schemes tailored to the specific preferences of each patient. As such, PROOF provides a balanced, patient-focused analysis of the improvement in function.

The integration of patient preferences into clinical outcomes is also used in the Schedule for the Evaluation of Individual Quality of Life (SEIQoL).^14^ In SEIQoL, patients express their preferences through a visual analog scale, determining how much each outcome should be weighted in the total score. By contrast, PROOF compares patients based on prioritization of each outcome individually. This preserves the outcome-specific information, which could be lost in a weighted total score. Hence, patient-reported preferences play a pivotal role, exerting a significant impact on the PROOF statistic. Different sets of preferences may be used, yet little is known about their reliability and consistency, which would be highly informative for future studies and refine the collection of patient-reported preferences.

In this study, therefore, we aim to assess the reliability, longitudinal changes, and prognostic value of different sets of patient preferences that make up the PROOF end point. This will provide important insight into how to refine the assessment of patient preferences and facilitate patient-focused drug development for ALS to address what matters most to patients.

## Methods

### Participants

Longitudinal data for this study were collected through The Netherlands ALS registry.^15^ In brief, patients diagnosed with possible, probable (laboratory-supported), or definite ALS according to the revised El Escorial criteria^16^ or those diagnosed with progressive muscular atrophy (PMA) or primary lateral sclerosis (PLS)^17^ have been registered centrally by The Netherlands ALS Center since 2006. Patients are identified by screening large hospital and specialized rehabilitation clinic registries, and by contacting Dutch neurologists. The capture rate of the registry is 70%–80% of all incident cases.^15^ Vital status is updated at quarterly intervals by checking the online municipal population register. Our study population included all surviving patients with ALS, PMA, or PLS who had participated in our previous study in June 2021,^10^ together with all newly diagnosed patients registered after June 2021. All patients alive on June 1, 2022, were approached by e-mail on June 8th 2022 and sent an online survey. A total of 1,648 diagnosed patients were alive in either June 2021 or June 2022 and potentially eligible for one of the surveys; 855 patients (52%) had previously provided consent to be approached and had a valid email address.

### Study Procedures

The online survey was constructed using a cloud-based clinical data management platform (Castor EDC, version 2022.1). The questionnaire consisted of a validated self-reported version of the revised ALS functional rating scale (ALSFRS-R).^18^ This consists of 12 items that can be clustered into 4 domains: (1) bulbar, items 1–3; (2) fine motor, items 4–6; (3) gross motor, items 7–9; and (4) respiratory functioning, items 10–12.^19,20^ In addition, the online questionnaire was supplemented with 2 questions to assess patient preferences (identical to those used in the 2021 survey):

- “Which domain bothers you the most?”
  - Bulbar domain
  - Fine motor domain
  - Gross motor domain
  - Respiratory domain
- “Imagine you will receive a treatment that delays disease progression, delay of which domain is the most important to you? Score the domain that is the most important to you with 1, and the domain that is least important with 4. If all domains are of equal importance to you, please select not applicable.”
  - Bulbar domain
  - Fine motor domain
  - Gross motor domain
  - Respiratory domain
  - Not applicable, all domains are of equal importance to me

A brief introduction was provided to outline which symptoms were associated with each domain. The questionnaire was repeated after 2 weeks to assess test-retest reliability. Automated data validations were programmed to minimize missing data and ensure data quality. In case of nonresponse, a one-time reminder was sent out after 10 days. The original questionnaire is available at tricals.shinyapps.io/PROOF/.

### Patient Preferences and Winning Rules

The questionnaire data produced 3 different sets of patient preferences: (1) the most bothersome domain (based on *question A*), (2) the most important domain (based on *question B,* i.e., the domain with rank 1), and (3) a fully ranked order of domain importance (based on all ranks of *question B*). Each of these 3 different sets of preferences can be used to define the order of the different domains of the ALSFRS-R to derive individual PROOF ranks. It should be noted that *question A* inquired about the domain that was most bothersome at the time of the questionnaire (hereafter referred to as “current” preferences), whereas *question B* related to a setting in the future (hereafter referred to as “future” preferences).

The exact scoring of PROOF and the method to derive patient ranks have been described elsewhere.^10^ In brief, PROOF compares each patient with all other patients. For each pair of patients, we determined which patient had the better outcome according to a set of *winning rules*. To illustrate, a “winning” patient is defined as the one who has the highest score on the most bothersome domain common to a pair of patients. These rules can be simply expanded, for example, to account for death (e.g., the “winning” patient is the one who survives, has the longest survival time, or, if both patients survive, has the highest score on the most bothersome domain, or if their most bothersome domains are the same, has the highest ALSFRS-R total score).^21^ We extended our online calculator to account for the different sets of patient preferences and to determine the winner in any pair of patients, given their preferences, domain scores, and survival time. Further examples are provided at tricals.shinyapps.io/PROOF/.

### Ranking Individual Patients

Regardless of the chosen set of preferences, each pairwise comparison results in an ordinal outcome: winner, loser, or tie. In these comparisons, a patient receives 1 point for a better outcome, 0.5 points for an equivalent outcome (tie), and 0 points for a worse outcome compared with the peer. By comparing a patient with all other patients in the study population, the sum of these comparisons produces an overall score. This score reflects how many patients have a worse or equal outcome to the individual. This process can be repeated for each patient in the study, after which we can rank patients based on their scores to determine their relative disease status in the study population. The patient with the lowest rank has the “worst disease status" while the one with the highest rank has the “best disease status." These ranks are valuable for group comparisons, such as determining whether the mean rank differs between treated and placebo groups, or for correlation with covariates. In addition, these ranks can serve as prognostic factors for individual patients in epidemiologic cohort research because they reflect the patients' percentile location in the population.

### Statistical Analysis

The objective of the analysis was 2-fold: (1) to assess the test-retest reliability of the individual PROOF ranks based on different sets of patient preferences and (2) to evaluate the association between PROOF ranks and survival time. Reliability of PROOF ranks was determined by linear mixed effects models—incorporating solely a random intercept—to estimate intraclass correlation coefficients (ICCs) for the ALSFRS-R domain scores, ALSFRS-R total score, and the individual patient ranks. Individual patient ranks were obtained by ranking patients based on the pooled data from both assessment times, thereby not only comparing each patient with all other patients at first assessment but also comparing each patient with themselves and with all other patients at week 2. In this way, one can assess whether a systematic change (i.e., bias) in ranking took place between the 2 assessment times and test potential differences using a paired *t*-test. The residual variance from the linear mixed model was further used to calculate the minimal detectable change (MDC) on an individual level (i.e., the difference between 2 measurements that is expected because of measurement error).^22^

Associations between patient preferences and survival time were explored by comparing survival distributions between PROOF-based ranks utilizing different sets of preferences. Kaplan-Meier curves were estimated for each quartile of ranks, thus dividing the sample size into 4 equal strata, ordered from the best (the top quartile) to the worst (the bottom quartile). In addition, we used Cox proportional hazard models to evaluate the association between survival and the ALSFRS-R and PROOF and to determine the hazard ratio for each quartile. As a sensitivity analysis, we replicated the analysis in a trial-eligible cohort, defined as having a diagnosis of ALS, a symptom duration ≤36 months, being younger than 80 years, and not using noninvasive ventilation at the time of survey. All codes and patient-level data are provided at tricals.shinyapps.io/PROOF/.

### Standard Protocol Approvals, Registrations, and Patient Consents

The medical ethics committee and institutional review board of the University Medical Center Utrecht (METC NedMec) approved this study (Study Registration Number: METC 22/522). Digital consent was obtained from all patients.

### Data Availability

The source code and patient-level data used in this study are available at tricals.shinyapps.io/PROOF/.

## Results

**Figure 1:**
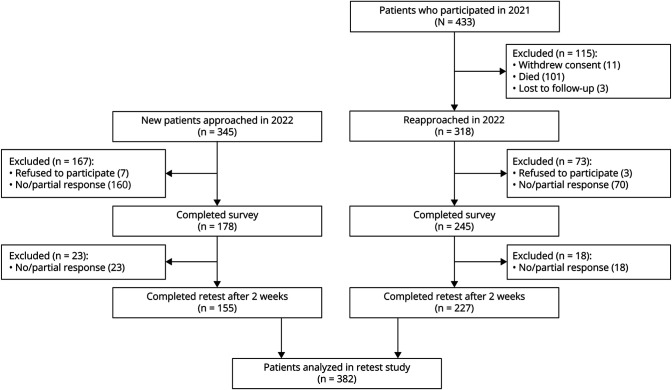
Flow Diagram of Patient Recruitment Patients who originally participated in 2021 were reapproached by email after 12 months together with 345 newly diagnosed patients. After 2 weeks, all responding patients received an invite to complete the same survey for a second time.

In total, 318 surviving patients of the 433 patients who participated in 2021 were reapproached by e-mail in June 2022, together with 345 newly diagnosed patients. A flow diagram of patient responses is depicted in Figure 1. Of the 663 patients who were approached in June 2022, 423 patients completed the survey (63.8%). After 2 weeks, the retest survey was completed by 382 of the 423 patients (90.3%); the median time between test and retest was 14 days (interquartile range 12–16). Caregiver assistance to complete the survey was required by 11.2% of the patients. We defined 2 analysis populations: (1) the reliability analysis population comprising those with complete test-retest data obtained in 2022 (N = 382) and (2) the survival analysis population comprising all 611 unique patients who have provided data at least once during either the 2021 (N = 433) or 2022 study (N = 178). Data on vital status were collected up to November 2023.

The baseline characteristics and patient-reported preferences of the different populations are provided in Table 1, together with the characteristics of the registry (source) population at the time of survey. As can be seen, when compared with all registry patients who were alive at time of survey, patients who participated in our study (N = 611) were younger and more often diagnosed with PMA or PLS. Preferences for the most bothersome domain (*current*) differed among noneligible vs trial-eligible patients (*p* < 0.001); trial-eligible patients expressed a preference for the bulbar domain more often, and for the respiratory domain less often, compared with noneligible patients. Likewise, trial-eligible patients indicated more often that they would prefer a specific domain to be improved by treatment (*future, p* = 0.022). The patient-reported preferences, stratified by MND subtype, are provided in eFigure 1, underscoring that the frequency distribution of what is most bothersome or important to patients will depend on the included study population in future settings.

**Table:** Patient Characteristics of the Enrolled Patient Populations

Patient characteristic	Registry (N = 1,648)	All patients (N = 611)	Reliability cohort (N = 382)	Trial-eligible patients (N = 174)
Age at baseline, y	67 (11)	65 (10)	65 (10)	64 (9)
Sex, male, n (%)	1,034 (63)	411 (67)	254 (67)	124 (71)
Site of symptom onset, bulbar, n (%)	367 (22)	105 (17)	57 (15)	41 (24)
Type of MND, n (%)				
ALS	1,117 (68)	387 (63)	233 (61)	174 (100)
PMA	254 (17)	102 (17)	62 (16)	0 (0)
PLS	277 (15)	122 (20)	87 (23)	0 (0)
Symptom duration,^a^ mo	42 (89)	44 (74)	44 (80)	19 (12)
ALSFRS-R total score	—	33 (10)	35 (9)	37 (7)
∆FRS, points per month	—	−0.40 (0.39)	−0.35 (0.35)	−0.58 (0.45)
Riluzole use, yes, n (%)	—	400 (66)	250 (65)	151 (87)
Gastrostomy, yes, n (%)	—	86 (14)	38 (10)	16 (9)
Respiratory support, yes, n (%)	—	121 (20)	62 (16)	0 (0)
Baseline patient preferences, n (%)				
Most bothersome domain (current)				
Bulbar	—	141 (23)	77 (20)	50 (29)
Fine motor	—	180 (29)	117 (31)	66 (38)
Gross motor	—	237 (39)	158 (41)	54 (31)
Respiratory	—	53 (9)	30 (8)	4 (2)
Most important domain (future)				
No preference (all equal)	—	243 (40)	141 (37)	57 (33)
Bulbar	—	123 (20)	85 (22)	41 (24)
Fine motor	—	45 (7)	32 (8)	15 (9)
Gross motor	—	67 (11)	38 (10)	13 (7)
Respiratory	—	133 (22)	86 (23)	48 (28)

Abbreviations: ALSFRS-R = ALS Functional Rating Scale; ∆FRS = ALSFRS-R—48/symptom duration; MND = motor neuron disease; PMA = progressive muscular atrophy; PLS = primary lateral sclerosis.

Data are expressed as mean (SD) or n (%). The trial-eligible patients are a subset of “all patients” and were defined as having ALS, a symptom duration ≤36 mo, being younger than 80 y, and not using noninvasive ventilation. The registry population was defined as all diagnosed patients who were alive in either June 2021 or June 2022 and potentially eligible to participate in the survey.

a Median (interquartile range).

### Test-Retest Reliability of Preferences

**Figure 2:**
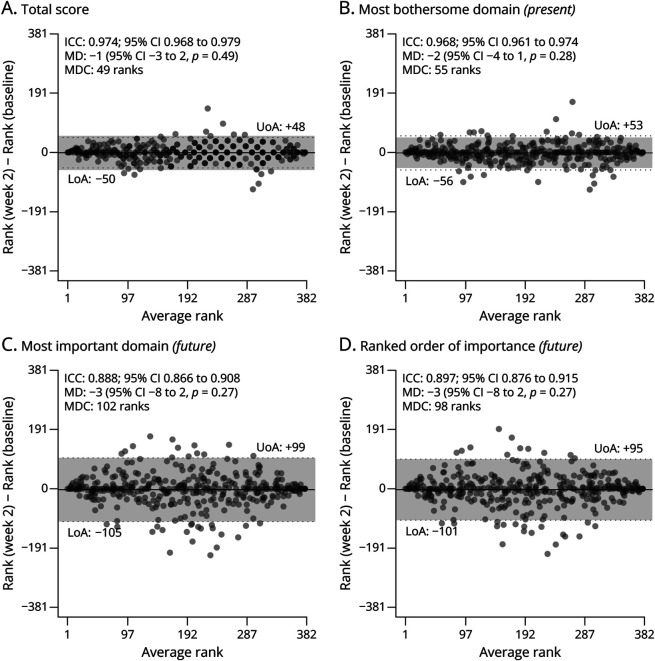
Bland-Altman Plot for Test-Retest Reliability of Patient Ranks Patients were ranked on 2 occasions (baseline and week 2) by using the ALSFRS-R total score (A), the most bothersome domain (B), the most important domain (C), or the ranked order of most important domains (D). The difference in ranks is provided on the y-axis, showing no systematic differences between baseline and week 2. ICC = intraclass correlation coefficient; MD = mean difference in ranks between week 2 and baseline; MDC = minimal detectable change (individual level); LoA = lower limit of agreement; UoA = upper limit of agreement.

The intrapatient reliability of PROOF ranks based on different sets of preferences is depicted in Figure 2. To allow for a direct comparison with the ALSFRS-R, ranking was also based on solely the ALSFRS-R total score. All versions of the PROOF ranking resulted in excellent reliability estimates with ICCs ranging from 0.89 to 0.97. PROOF ranks based on the most bothersome domain (*current*) had virtually the same reliability as the ALSFRS-R total score despite the increase in number of unique patient ranks (i.e., there were 46 unique ranks for the ALSFRS-R total score vs 489 unique ranks for PROOF). Significantly, none of the PROOF versions had systematic biases between baseline and week 2 (mean rank differences between baseline and week 2, all *p* > 0.20). Measures of reliability for the ALSFRS-R subdomains are provided in eTable 1; these were similar to those reported previously.^18^ The same ALSFRS-R subdomain scores were reported by 59.7% (bulbar), 50.5% (fine motor), 51.8% (gross motor), and 69.9% (respiratory) of the patients.

Ranks based on the most important domain (*future*) and the ranked order of domains (*future*) had an increase in individual rank variability, resulting in relatively lower ICCs and larger MDCs. These differences were driven by a difference in reproducibility of preferences: of the 382 patients, 299 (78.3%) reported the same most bothersome domain (*current*) on both occasions, 237 (62.0%) reported the same most important domain (*future*), and 186 (48.7%) replicated an identical ranked order for the entire domain importance (eFigure 2). Cognitive status of 285 patients was available at diagnosis, 22 of whom (8%) had cognitive impairment based on the Edinburgh Cognitive and Behavioural ALS Screen.^23^ There was no statistically significant difference in reproducibility by the presence of cognitive impairment (with vs without impairment): most bothersome domain (*current*) 81.8% vs 76.8%, *p =* 0.79; most important domain (*future*) 50.0% vs 62.0%, *p =* 0.36; ranked order of domain importance (*future*) 45.5% vs 47.1%, *p* > 0.99. Patients with cognitive impairment were, however, significantly more often assisted by caregivers than those without cognitive impairment (27.3% vs 9.5%, *p* = 0.0218). On the whole, help from a caregiver did not significantly affect reproducibility (all *p* > 0.25), nor was there a statistical difference between trial-eligible and ineligible patients (*results not shown*).

### Longitudinal Changes in Preferences and Survival

**Figure 3:**
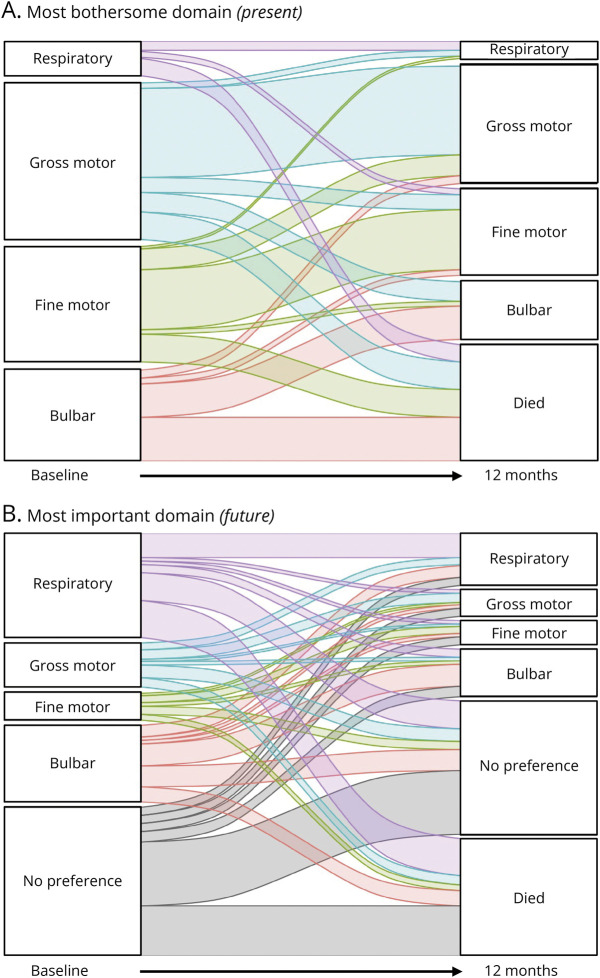
Longitudinal Changes in Patient Preferences During follow-up, 101 of the 433 patients died between baseline and month 12; a follow-up measurement was available for 245 of the 332 surviving patients. (A) Longitudinal change from baseline preference for the most bothersome domain (*present*); (B) longitudinal change from baseline preference for the most important domain (*future*).

For the initial 2021 cohort, 101 of the 433 patients died between baseline and month 12; a 12-month follow-up measurement was available for 245 out of the 332 surviving patients (73.8%). The longitudinal changes in patient preferences are provided in Figure 3, illustrating the change in preferences over time, especially for future events. Of the 245 patients alive at month 12, 67.8% reported the same most bothersome domain (*current*) at baseline and month 12%, and 44.5% reported the same most important domain (*future*). As these metrics are lower than the 2-week reproducibility data (i.e., 78.3% and 62.0%, respectively, both *p* < 0.001), an additional longitudinal component likely induces change in preferences as disease progresses. Moreover, the preference at baseline for the most bothersome domain (*current*) was strongly associated with the probability of being alive at month 12 (Figure 3A, *p <* 0.001), which was less apparent for the most important domain (*future,* Figure 3B, *p =* 0.096). This is perhaps unsurprising given the association between the most bothersome domain and actual domain scores,^10^ and the differential impact of each domain on overall survival.^8^

**Figure 4:**
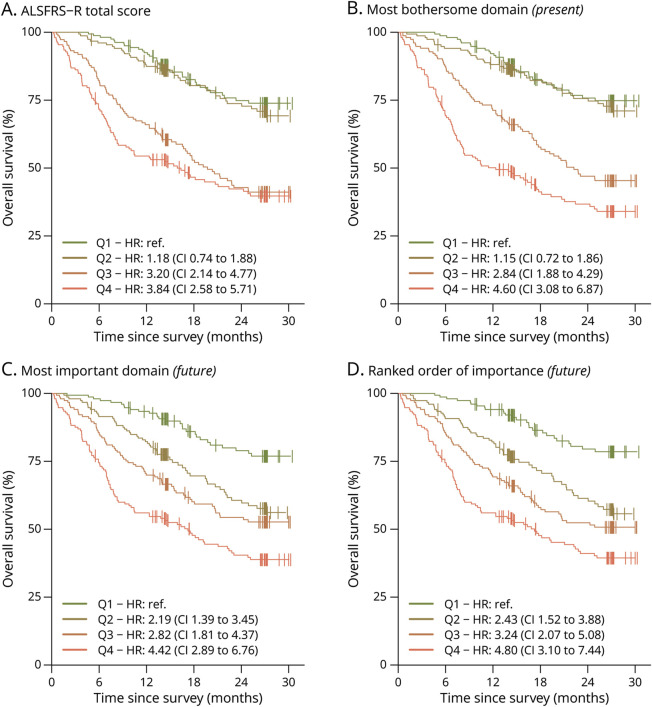
Cumulative Survival Stratified by Baseline Rank Overall survival was stratified by rank into 4 equal quartiles (N = 611): 1–25th percentile (*Q4*), 26–50th percentile (*Q3*), 51–75th percentile (*Q2*), and 76–100th percentile (*Q1*). Ranks were based on the ALSFRS-R total score (A), the most bothersome domain (B), the most important domain (C), or the fully ranked order of importance (D). As can be seen, adding the patient-reported preferences may increase the contrast in overall survival between groups.

Overall, 240 of the 611 patients died during follow-up. The cumulative survival stratified by rank at baseline is presented in Figure 4. PROOF-based ranks led to strong predictive abilities among all ranking systems for overall survival (hazard ratios per 10th rank percentile ranged from 0.80 to 0.83 [95% CI range 0.76–0.87], all *p* < 0.001; eTable 2) and a potential increased separation between rank-stratified subgroups (Figure 4, C and D). Note the ability to separate the survival experience between the 4 quartile groups for PROOF in comparison with those based on solely the ALSFRS-R. Using the ranked order of importance, there was a nearly linear increase in hazard compared with the first quartile, with hazard ratios increasing from 2.43 (95% CI 1.52–3.88) for the second quartile, 3.24 (95% CI 2.07–5.08) for the third quartile and to 4.80 (95% CI 3.10–7.44) for the fourth quartile (all *p* < 0.001). Similar results were found when the analysis was restricted to trial-eligible patients (eFigure 3 and eTable 3).

## Discussion

In this study, we evaluated the consistency of patient preferences and their association with survival in a large cohort of patients with ALS. We show how patient-reported preferences can be measured and integrated reliably with a conventional clinical end point without leading to systematic bias. Patient preferences may provide unique prognostic information in addition to what is already measured by the ALSFRS-R. Hence, by combining end points with patient-reported preferences, one could obtain a better reflection of individual disease severity and the patient-perceived disease impact, which would be highly valuable for clinical trials. Patient-reported preferences do associate with survival outcome and evolve over time. Conducting repeated assessments in clinical trials will be essential to gain a comprehensive understanding of how a drug effectively addresses patients' concerns and improves what matters most to them.

Our results, supported by previous work,^7,10^ outline the wide variability between patients in their most bothersome symptoms and in perceived preferences regarding future events. As was noted previously,^7^ not all symptoms—or loss in functional domains—have an equal impact on the patient's life. Hence, summing different symptoms or affected domains into overall composite scores, like the ALSFRS-R total score, without considering the differential significance of each domain to individual patients, may not accurately reflect disease severity or the impact of disease on the patient's life. When used in clinical trials, this could create a skewed representation of the (perceived) treatment benefit. This is particularly noteworthy when considering that not all patients will develop all symptoms^24^ and that treatments could benefit each domain divergently.^25^

These findings support the concept that—to achieve better clinical outcome measures and patient-focused approaches to clinical trials—it will be essential to recognize the value of patient-reported preferences in discriminating the impact on each domain and incorporate these into outcome evaluations. The framework used by PROOF is straightforward and a well-validated statistical strategy to account for differential domain importance.^9,12^ A major benefit of PROOF is that it results in a single overall assessment of the drug's effect on functional status weighted by the preferences of each individual patient in the study. An important caveat is, however, that incorporating patient-reported preferences will add an additional layer of variability, including differences in preferences, but also in how the individual preferences are collected; this requires mitigation because of the potential to decrease outcome sensitivity.

Based on our study, we have identified a few key challenges that should be considered. First, within-patient variability in patient-reported preferences about future events was increased compared with their reported preferences about current events. Inquiring about future events may not be congruent with actual lived experience, making it complex for a patient to accurately foresee the potential impact if symptoms were to develop. Second, simultaneously ranking multiple disease concepts is challenging. By contrast, choosing only 1 (most important) aspect might be too simplistic for a multifaceted disease such as ALS. This is further complicated by preferences changing over time and the potential involvement of cognitive disabilities at later stages of the disease.^26,27^

The impact of cognition poses an unresolved challenge. In our study, we found no substantial differences in reproducibility between individuals with and those without known cognitive impairment, although cognitive re-evaluation was not conducted at the time of survey. Remote (self-reported) cognitive assessments are unfortunately not yet widely available,^28^ and validation of patient-reported preferences may need to be repeated in an in-clinic setting along with cognitive reassessment, for example, as a substudy within a clinical trial. Furthermore, patients with cognitive impairment, or those with advanced disease, are more likely to receive a higher degree of caregiver assistance,^29^ with the caregiver potentially completing the survey. As such, a nontrivial task will be to evaluate the concordance between caregiver-reported and patient-reported preferences.

Based on these limitations, a few recommendations can be made to improve the assessment of patient-reported preferences. Assessment should preferably be congruent with actual lived experience, thereby prioritizing domains that currently most bother the patient. Second, providing patients with multiple binary questions—e.g., choosing the most affected domain among varying combinations of 2 domains—may help to lower complexity (i.e., similar to a discrete-choice experiment).^30^ One could consider inquiring about domain preferences or, alternatively, about preferences for the individual symptoms that make up the domains. The latter may be preferable because whether symptoms that are part of the same domain have an equal impact on the patient's life (e.g., speech vs swallowing difficulties in the bulbar domain) might be contested. Moreover, preferences should be reassessed at regular intervals aligned with the collection of key clinical outcomes. As such, domains or outcomes can be reweighted according to current preferences at each assessment time. This would also facilitate the interpretation of PROOF in clinical trials as PROOF would reflect the treatment benefit for those domains that are currently most significant to patients, ultimately resulting in a potentially more clinically relevant effect measure.

Nevertheless, defining a clinically important effect size for PROOF presents challenges owing to the nonparametric nature of the scale. One solution involves incorporating a minimally clinically important difference (MCID) into the PROOF algorithm, scoring a patient as a “winner” only when the difference with the comparator exceeds a certain threshold.^9^ As such, PROOF reflects the probability that a treated patient has a better outcome—by at least the MCID—than a placebo patient. Estimating MCID for each outcome used by PROOF would be of paramount importance. In addition, linking changes in PROOF-based ranks to alterations in quality of life, overall survival and patient characteristics would further facilitate the clinical interpretation of PROOF. External validation in prospective longitudinal studies, preferably conducted in diverse international patient populations and clinical trial settings, is necessary to ultimately build toward a formal qualification assessment by the major regulatory agencies.

In conclusion, in this study we have shown how patient-reported preferences can be measured and integrated reliably with a conventional clinical end point without introducing systematic bias. Incorporating patient-reported preferences may result in a better metric of individual disease severity and could facilitate a more patient-focused assessment of novel treatments. An important caveat is that incorporating patient-reported preferences will add an additional layer of variability, which potentially reduces outcome sensitivity. Making assessments congruent with actual lived experience, binary and repeated over time, might improve the assessment. Ultimately, this may further extend our understanding of the patient's concerns and lead to the development of treatments that address what matters most to patients.

## Appendix Authors

**Table:**

Name	Location	Contribution
Ruben P.A. van Eijk, MD, PhD	UMC Utrecht Brain Center, University Medical Center Utrecht	Drafting/revision of the manuscript for content, including medical writing for content; major role in the acquisition of data; study concept or design; analysis or interpretation of data
Leonard H. van den Berg, MD, PhD	UMC Utrecht Brain Center, University Medical Center Utrecht	Drafting/revision of the manuscript for content, including medical writing for content; major role in the acquisition of data
Ying Lu, PhD	Department of Biomedical Data Science, Stanford University	Drafting/revision of the manuscript for content, including medical writing for content; major role in the acquisition of data; study concept or design; analysis or interpretation of data

## Glossary

ALS: amyotrophic lateral sclerosis
ALSFRS-R: ALS functional rating scale
ICC: intraclass correlation coefficient
MCID: minimally clinically important difference
MDC: minimal detectable change
PLS: primary lateral sclerosis
PMA: progressive muscular atrophy
PROOF: Patient-Ranked Order of Function
SEIQoL: Schedule for the Evaluation of Individual Quality of Life
